# Role of lactate dehydrogenase A in the regulation of podocyte metabolism and glucose uptake under hyperglycemic conditions

**DOI:** 10.1038/s41598-025-98797-0

**Published:** 2025-04-23

**Authors:** Audzeyenka Irena, Grochowalska Klaudia, Szrejder Maria, Kulesza Tomasz, Rachubik Patrycja, Rogacka Dorota, Piwkowska Agnieszka

**Affiliations:** 1 Laboratory of Molecular and Cellular Nephrology, Mossakowski Medical Research Institute, Polish Academy of Sciences, Gdańsk, Poland; 2 Laboratory of Molecular Enzymology and Oncology, Intercollegiate Faculty of Biotechnology, Medical University of Gdansk, Gdansk, Poland

**Keywords:** Lactate dehydrogenase, Hyperglycemia, Insulin, Glycolysis, Glucose uptake, Lactate, Cell biology, Physiology

## Abstract

Lactate is a cellular product of glycolytic metabolism, serving as both an additional oxidative energy substrate and a signaling molecule in metabolic regulation. Plasma lactate levels are elevated in diabetes, and chronic extracellular lactic acidosis is recognized as a negative prognostic marker for the disease. The development of diabetic kidney disease is closely associated with podocyte injury, which forms a crucial layer of the glomerular filtration barrier. Given that high extracellular glucose concentrations also induce lactate production and excretion in podocytes, we hypothesize that an appropriate LDH expression pattern is crucial for maintaining proper podocyte metabolism and function. Our research shows that hyperglycemia significantly decreases lactate dehydrogenase activity in podocytes. Specifically, reduced LDHA expression under hyperglycemic conditions contributes to metabolic disturbances in these cells. Lower LDH activity results in decreased glycolytic activity, altered expression of monocarboxylate transporters, reduced insulin-dependent glucose uptake, and a decrease in the number of podocyte foot processes. These findings underscore the essential role of LDHA in the metabolic adaptation of podocytes to elevated glucose levels typical of diabetes. By elucidating the molecular mechanisms underlying podocyte injury, our study provides new insights into potential therapeutic targets for preventing or mitigating diabetic kidney disease.

**Supplementary Information:**

The online version contains supplementary material available at 10.1038/s41598-025-98797-0.

## Introduction

Diabetic kidney disease (DKD) and its most severe form, end-stage renal disease, are significant causes of a shorter lifespan in people with diabetes. According to the International Diabetes Federation Diabetes Atlas (10th edition), nearly 550 million people worldwide were living with diabetes in 2021. This number is projected to increase by 25% by 2030 and by 51% by 2045^1^.

The glomerulus is one of the major site of diabetic injury in the kidney. Glomerular hypertrophy and podocyte depletion are glomerular hallmarks of progressive DKD, and the degree of podocyte loss correlates with disease severity. Podocytes are highly specialized cells that wrap around glomerular capillaries and comprise a key component of the glomerular filtration barrier. The podocyte cell body gives rise to primary processes that branch into foot processes; in turn, foot processes of neighboring podocytes establish a highly branched, interdigitating pattern, known as a slit diaphragm^2–4^. Podocyte slit diaphragms are the target of injury in many glomerular diseases, including arterial hypertension, inflammation, and diabetes mellitus. The loss of podocyte function causes changes in kidney structure and function that resemble renal complications in human diabetes^5,6^. Podocytes were recently shown to rely primarily on anaerobic glycolysis and only to a minor extent on the β-oxidation of lipids^7^. Moreover, mitochondrial oxidative phosphorylation has only a limited impact on overall podocyte adenosine triphosphate synthesis^7,8^. Other authors reported that a high glucose concentration forced podocytes to switch from mitochondrial oxidative phosphorylation to glycolysis, resulting in lactic acidosis^9^.

Evidence increasingly suggests that lactate is not merely a metabolic byproduct; it can be favored over glucose as an energy source and function as an important metabolic reserve molecule^10^. We recently identified the presence of lactate transporters in podocytes^11^. Furthermore, we observed changes in the expression of these transporters in response to glucose deprivation and lactate supplementation. Exposure to lactate preserved podocyte survival under glucose-restricted conditions^11^. We also clearly showed the colocalization of monocarboxylate transporter 1 (MCT1) and MCT2 within mitochondria. The mitochondrial localization of both transporters was particularly pronounced in cells that were supplemented with lactate^12^. Moreover, lactate stimulation changed mitochondrial dynamics and respiratory efficiency in primary rat podocytes. Another important finding was that glucose deprivation or lactate supplementation significantly increased the oxygen consumption rate/extracellular acidification rate ratio, indicating a transition toward greater reliance on oxidative phosphorylation for cellular energy generation^12^. Thus, podocytes utilize lactate as an energy substrate and have a sophisticated system to maintain lactate homeostasis, underscoring its vital role in podocyte metabolism, particularly during energy fluctuations.

Lactate dehydrogenase (LDH) is a key enzyme that is responsible for maintaining the balance between intracellular concentrations of lactate and pyruvate, depending on the cell’s energy requirements. Lactate dehydrogenase A (LDHA) catalyzes the conversion of pyruvate to lactate, and lactate dehydrogenase B (LDHB) catalyzes the reverse reaction. Previous research confirmed the presence of LDH in the rat kidney^13^. The exact cellular localization of these isoforms was recently identified along the nephron tubule^14^. According to Osis et al., LDHA is predominantly expressed in proximal regions, and LDHB is expressed in distal regions. The expression of these isoforms has also been shown to be significantly altered in models of acute kidney injury and chronic kidney disease^14^. Our recent study was the first to demonstrate the presence and specific localization of both LDH isoforms in podocytes^12^.

In the present study, we found that expression of the LDHA isoform is altered in a hyperglycemic environment. We showed that lower expression of lactate dehydrogenase A is responsible for metabolic disturbances in podocytes under hyperglycemic conditions, such as decreased glycolytic activity and altered expression of monocarboxylate transporters. We also found that silencing LDHA expression results in the inhibition of cellular glucose transport and a reduction of the amount of nephrin, a key slit diaphragm marker protein in podocytes. This highlights the crucial role of this LDHA isoform in the cellular metabolic adaptation to high glucose levels and in the regulation of insulin-dependent glucose uptake in cultured rat podocytes.

## Results

### Hyperglycemia and oxamate affected LDH expression pattern in podocytes

**Fig. 1:**
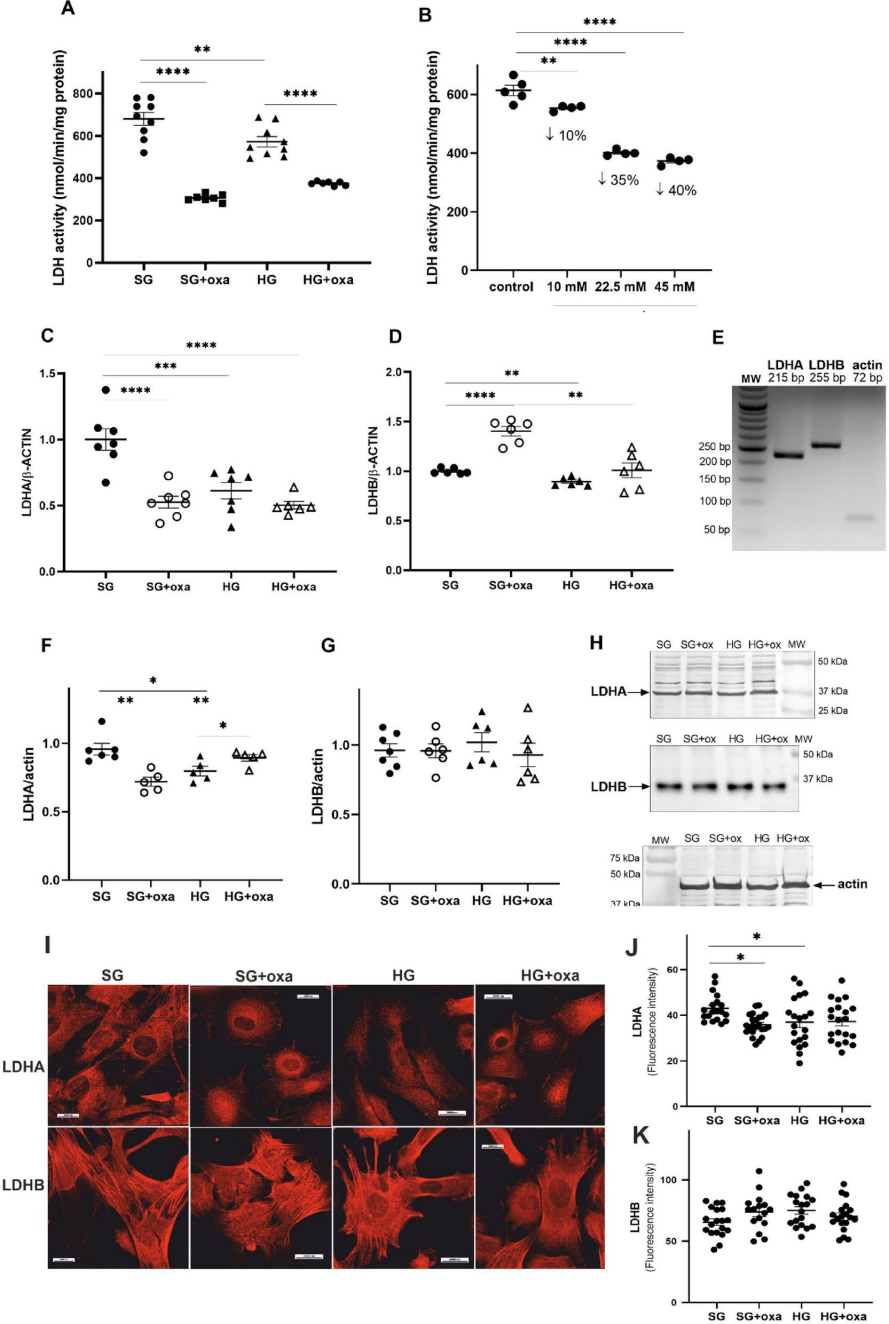
Hyperglycemia affected the activity, expression and protein level of LDH in cultured rat podocytes. Cells were treated with 45 mM oxamate in the presence of SG and HG medium for 5 days. (**A**) Lactate dehydrogenase activity was measured using the enzymatic method. (**B**) The influence of various concentrations of oxamate on LDH activity in podocytes that were cultured in a standard glucose concentration for 24 h. The influence of HG or oxomate on mRNA expression of LDHA (**C**) and LDHB (**D**). (**E**) Representative 2.5% agarose gel of PCR products for LDHA, LDHB, and β-actin. Cell lysates were analyzed by immunoblotting using anti-LDHA (**F**) and anti-LDHB (**G**). (**H**) Representative blot image. All data in this figure were analyzed using ANOVA. (**I**–**K**) Influence of high glucose concentration and oxamate on cellular distribution of both LDH isoforms in primary rat podocytes. Podocytes were immunostained with anti-LDHA or anti-LDHB antibody as described in the Methods section. oxa, oxamate. When the overall *F* tests showed significance, *post hoc* comparisons were conducted using Tukey’s adjustment method to identify specific pairwise differences that were significant. *p < 0.05, **p < 0.01, ***p < 0.001, ****p < 0.0001.

In the present study, we found a decrease in LDH activity in podocytes that were exposed to a HG concentration (Fig. 1A). To investigate the role of LDH activity on podocyte metabolism, we used an inhibitor of LDH, oxamate. Figure 1B shows the dose-dependent influence on LDH activity in podocytes. In the present study, we used 45 mM oxamate, which induced an approximately 40% decrease in LDH activity in podocytes. This concentration was in accordance with another study [22].

**Fig. 2:**
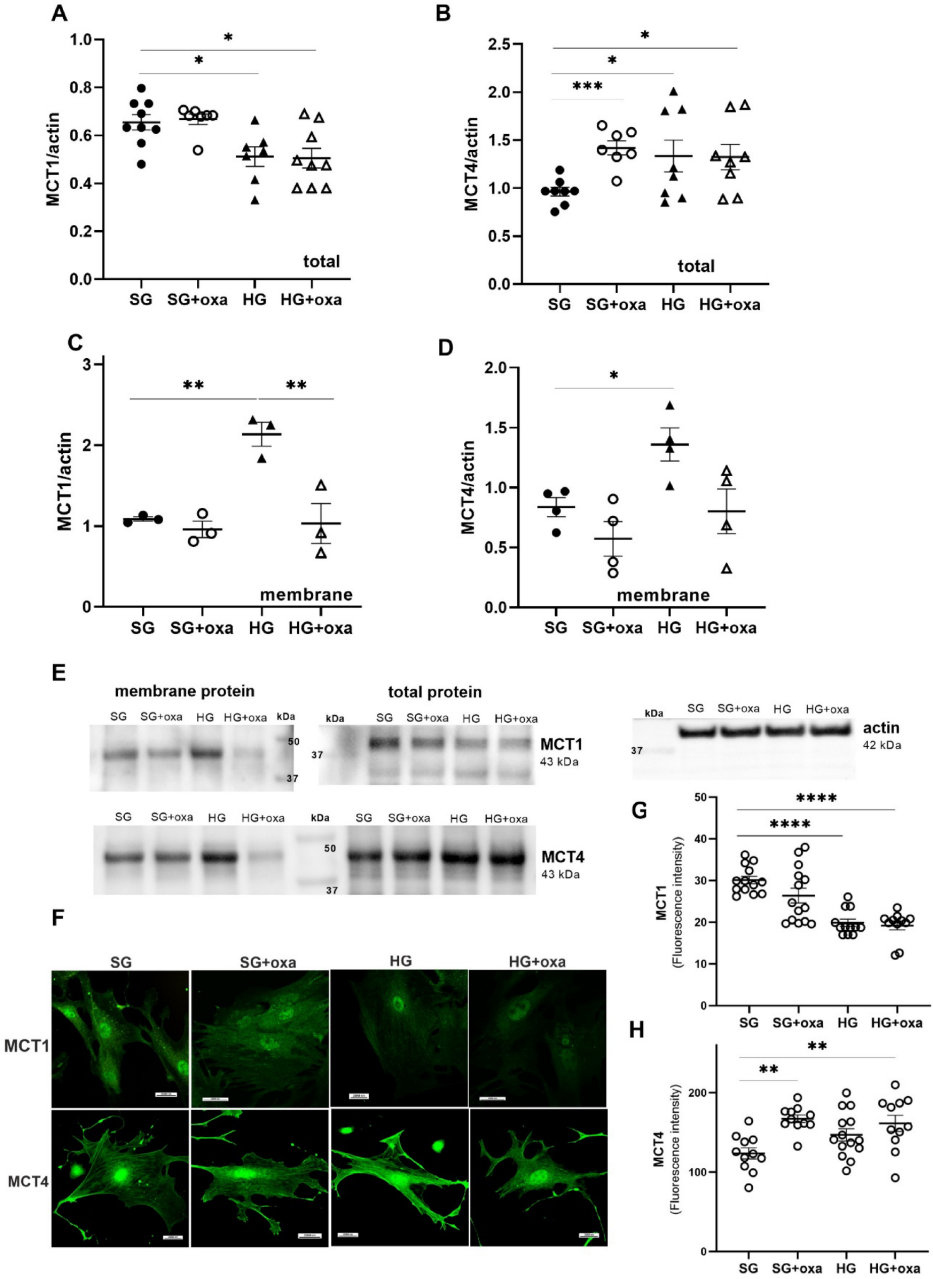
Expression and localization of MCT1 and MCT4 in podocytes cultured under high glucose concentration. (**A**–**D**) Western blot analyses of the total expression (A, B) and membrane expression (C, D) of MCT1 and MCT4 in the present of HG (30 mM) and after oxamate stimulation (45 mM, 24 h). (**E**) Representative immunoblot image. (**F**–**H**) Confocal microscopy showing changes in MCT1 and MCT4 signal patterns in primary rat podocytes that were exposed to oxamate (45 mM) or cultured in HG. All data in this figure were analyzed using ANOVA. When the overall *F* tests showed significance, *post hoc* comparisons were conducted using Tukey’s adjustment method to identify specific pairwise differences that were significant. *p < 0.05, **p < 0.01, ***p < 0.001.

In previous studies, we confirmed the presence of two LDH isoforms in podocytes^12^. In the next step of our research, we investigated the influence of a hyperglycemic environment on LDH isoform expression in podocytes (Fig. 1C,D). We found that HG significantly decreased expression of the LDHA and LDHB isoforms by approximately 39% and 11%, respectively. Changes in expression that were induced by a HG concentration translated only to a lower amount of LDHA protein (17%, *p* = 0.0194; Fig. 2F). This indicated that the lower LDH activity that was observed in HG was attributable to a lower amount of LDHA protein in podocytes. Moreover, we found that the inhibition of LDH activity by oxamate mainly decreased the expression and protein level of the LDHA isoform (Fig. 1). We also found that HG or oxamate changed the intracellular localization of LDHA (Fig. 1I–K). We detected its disappearance in perimembrane areas and processes.

### Expression and distribution of MCT1 and MCT4 in podocytes cultured in high glucose concentration

Because the major product of LDHA activity is lactate, we examined the expression and cellular distribution of two major lactate transporters, MCT1 and MCT4, under the investigated conditions. Monocarboxylate transporters are bidirectional symporters that are sensitive to trans-stimulation by lactate and hydrogen ion gradients^15^. Moreover, other studies suggest that MCT1 and MCT4 are primarily responsible for lactate uptake from the circulation and lactate extrusion out of the cell, respectively^16^. We showed that HG decreased the expression of MCT1 and increased MCT4 in podocytes. We observed the same effect after the inhibition of LDH activity with oxamate (Fig. 2A,B). This indicates higher expression of the transporter that is responsible for lactate efflux from the cell, which is observed in cells with enhanced glucose metabolism. Moreover, we identified a correlation between the inhibition of LDH activity and the expression pattern of both lactate transporters. We also found that a hyperglycemic environment increased levels of both transporters in the cell membrane (Fig. 2C,D). We confirmed this pattern using immunofluorescence, in which we also observed higher levels of both transporters near the cell membrane (Fig. 2F). However, oxamate only in the presence of HG caused a reduction of the membrane localization of both transporters.

### Glycolytic flux decreased in primary rat podocytes exposed to oxamate

**Fig. 3:**
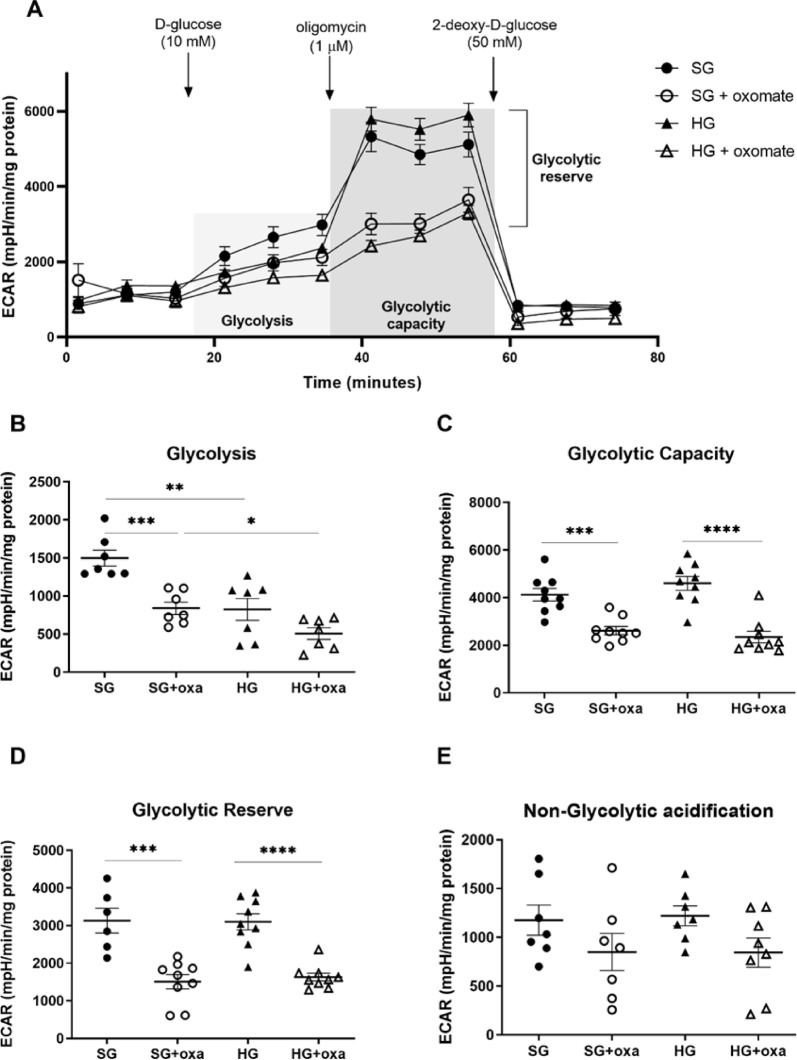
Glycolytic flux is altered in oxamate-treated primary rat podocytes. We estimated the efficiency of glycolysis based on the ECAR using a Seahorse analyzer. (**A**) Extracellular acidification rate of podocytes after oxamate stimulation (45 mM, 24 h) and cultured in HG (5 days) after a subsequent injection of glucose, oligomycin, and 2-deoxy-D-glucose. The final concentration of glucose was 10 mM. The final concentration of oligomycin was 1 µM. The final concentration of 2-deoxy-D-glucose was 50 mM. (**B**) Effects of oxamate and HG on glycolysis in podocytes. *p < 0.05, **p < 0.01, ***p < 0.001, Tukey’s test. *n* = 7. (**C**) Glycolytic capacity was decreased by oxamate. ***p < 0.001, ****p < 0.0001, Sidak’s test. *n* = 9. (**D**) Oxamate decreased glycolytic reserve ([glycolytic capacity] – [glycolysis]) in podocytes. ***p < 0.001, Sidak’s test. *n* = 6–9. (**E**) Non-glycolytic acidification in podocytes treated with oxamate or cultured in HG (*n* = 7–8). oxa, oxamate.

Energy metabolism in podocyte foot processes is primarily sustained by glycolysis because mitochondria are too large to fit into the lumen of the smallest processes^8^. Therefore, in the next step, we investigated the role of LDH in the regulation of glycolytic flux in podocytes using the real-time recording of ECARs. We showed that hyperglycemic conditions significantly decreased glycolysis (by 45%, *p* = 0.0026) but did not influence glycolytic capacity, glycolytic reserve, or non-glycolytic acidification (Fig. 3). Moreover, the inhibition of LDH activity by oxamate decreased glycolysis (by 44% vs. SG, *p* = 0.0003; by 38% vs. HG, *p* = 0.0769; Fig. 3B), glycolytic capacity (by 37% vs. SG, *p* = 0.0002; by 49% vs. HG, *p* < 0.0001; Fig. 3C), and glycolytic reserve (52% vs. SG, *p* = 0.0002; by 47% vs. HG, *p* < 0.0001; Fig. 3D) in podocytes, but it did not affect non-glycolytic acidification (Fig. 3E). These results indicate that LDH is an important regulator of the glycolytic pathway in these cells.

### Influence of oxamate on pyruvate kinase activity and lactate, pyruvate, and glycogen levels

**Fig. 4:**
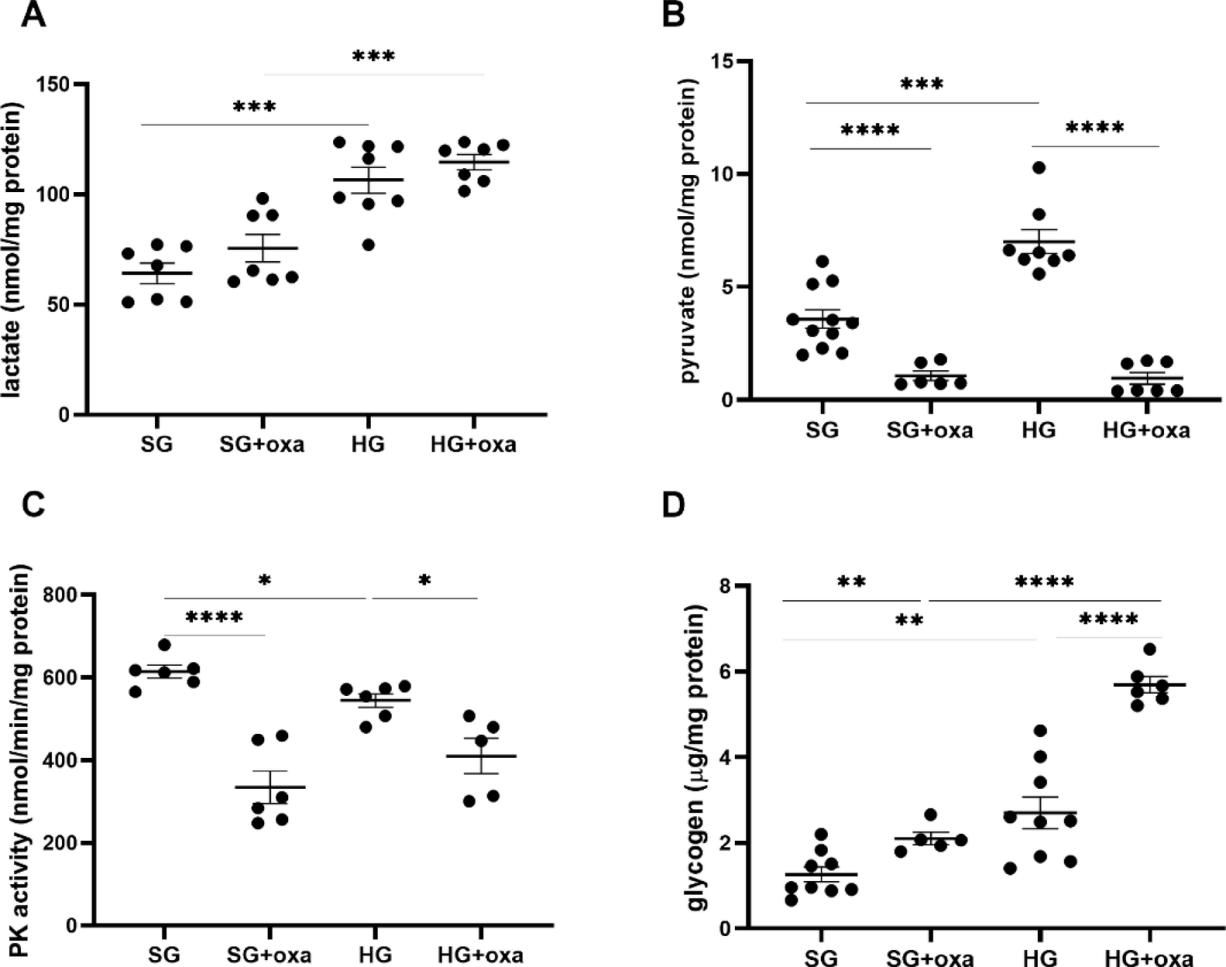
Influence of hyperglycemia and LDH inhibition on pyruvate kinase activity and intracellular concentration of lactate, pyruvate, and glycogen in podocytes. Cells were incubated in standard glucose (*SG*) or high glucose (*HG*) medium in the presence or absence of oxamate (oxa; 45 mM, 24 h). The amount of lactate (**A**), pyruvate (**B**), and glycogen (**D**) was measured in cell lysates using commercial tests. (**C**) Pyruvate kinase (PK) activity was analyzed using enzymatic methods. All data in this figure were analyzed using ANOVA. When the overall *F* tests showed significance, *post hoc* comparisons were conducted using Tukey’s adjustment method to identify specific pairwise differences that were significant. *p < 0.05, **p < 0.01, ***p < 0.001, ****p < 0.0001.

After 5 days of incubation in HG or after oxamate treatment, we observed an adaptive decrease in glucose metabolic flux. These changes were accompanied by increases in the amount of intracellular lactate, pyruvate, and glycogen (Fig. 4). We also found that 5 days of incubation with HG decreased the activity of pyruvate kinase (from 614.7 ± 15.6 to 545.0 ± 16.7 nm/min/mg protein, *p* = 0.0124; Fig. 4C). One important finding of our study was that oxamate also reduced pyruvate kinase activity under both SG and HG conditions by ~ 45% (*p* < 0.0001) and ~ 25% (*p* = 0.0124), respectively. This finding provided further evidence that LDH is an important regulator of glycolysis in podocytes. Along with inhibiting the metabolic flux of glucose, oxamate also increased the intracellular accumulation of lactate and glycogen (Fig. 4A,D) and significantly decreased the amount of pyruvate (Fig. 4B).

### Effect of LDH inhibition on glucose uptake in primary rat podocytes

**Fig. 5:**
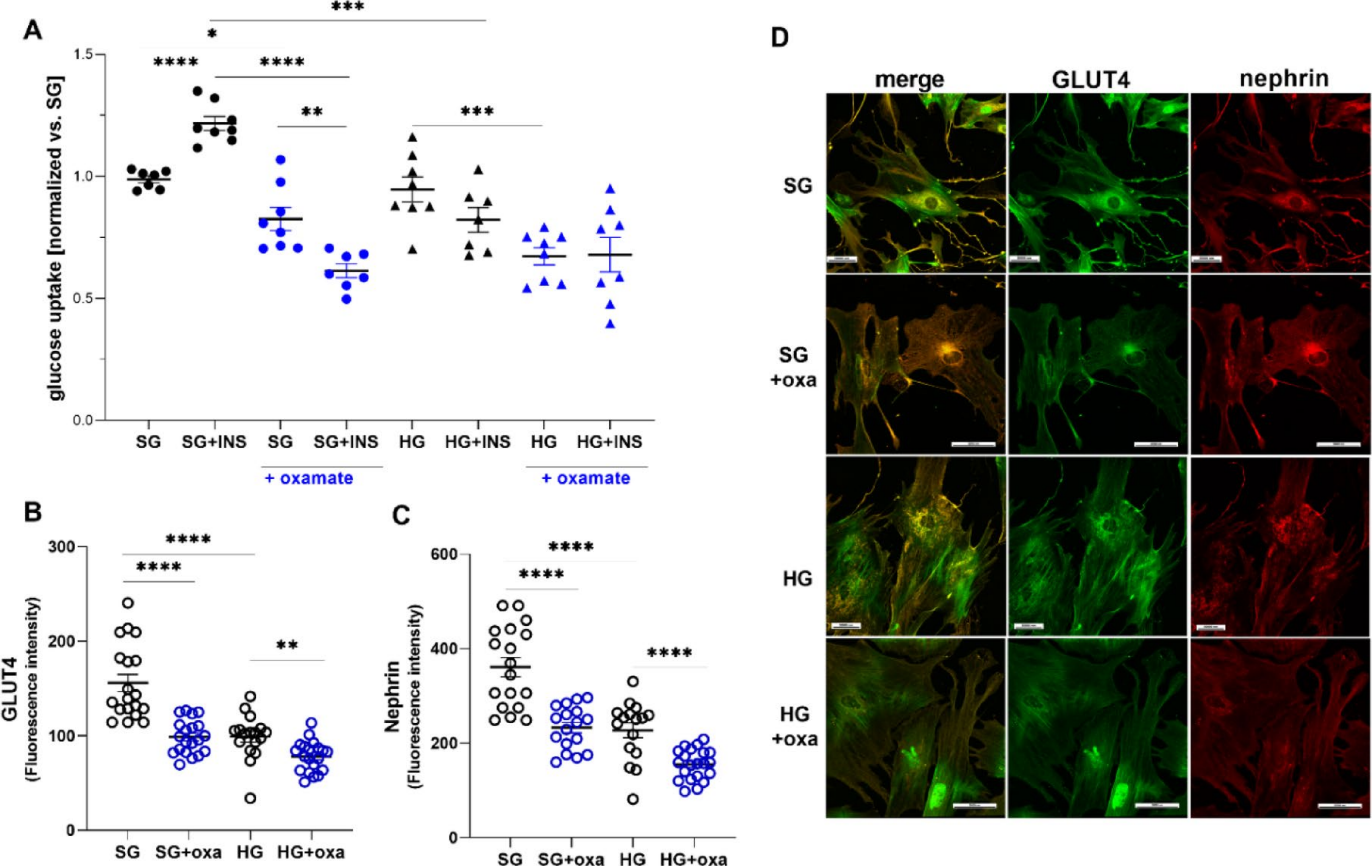
Role of LDH inhibition in insulin-dependent glucose uptake in primary rat podocytes. (**A**) Basal and insulin-dependent glucose uptake was measured. Glucose uptake measurements began with the addition of 0.5 µCi of (1,2-3* H*)-deoxy-D-glucose diluted in non-radioactive glucose to final concentrations of 50 µM and 300 nM insulin (INS). Glucose uptake was measured for 10 min. (**B**–**D**) Influence of oxamate on GLUT4 (B) and nephrin (C) levels and the colocalization of both proteins in primary rat podocytes (D). The mean fluorescence intensity of the outlined regions of interest was measured using Nikon NIS-Elements software with the Automated Measurement Results option. All data in this figure were analyzed using ANOVA. When the overall *F* tests indicated significance, *post hoc* comparisons were conducted using Tukey’s adjustment method to identify specific pairwise differences that were significant. *p < 0.05, **p < 0.01, ***p < 0.001, ****p < 0.0001.

In our previous studies, we showed that 5 days of incubation in HG induced insulin resistance in podocytes^17,18^. To investigate whether LDH participates in the regulation of glucose uptake in podocytes, cells were treated with oxamate for 24 h. The effect of oxamate on basal and insulin-dependent glucose uptake in podocytes that were cultured under HG conditions was compared with cells that were cultured under SG conditions. In control podocytes, we observed a 22% increase in insulin-dependent glucose uptake (*p* < 0.0001; Fig. 5A). After treatment with oxamate, a significant decrease in basal glucose uptake was observed in both control cells (17%, *p* = 0.089) and HG-cultured cells (29%, *p* = 0.0006). Moreover, the insulin response in the presence of oxamate was attenuated in control cells (Fig. 5A). Along with disturbances in intracellular glucose transport, we observed changes in the localization and quantity of two key proteins that are essential for proper insulin signaling in podocytes. Previous studies showed that alterations of the amount and localization of GLUT4 and nephrin are responsible for insulin resistance in podocytes^19,20^. The present study confirmed this relationship. We also demonstrated that the inhibition of LDH activity in control cells reduced levels of both proteins to levels that were observed in cells that were exposed to HG conditions (Fig. 5B,C). The impact of oxamate on podocyte morphology is also significant. We found that in the presence of this inhibitor, there was a loss of podocyte foot processes (Fig. 5D). Thus, LDH is an important regulator of glucose uptake in podocytes.

### Downregulation of LDHA attenuated the effect of insulin on glucose uptake in podocytes

**Fig. 6:**
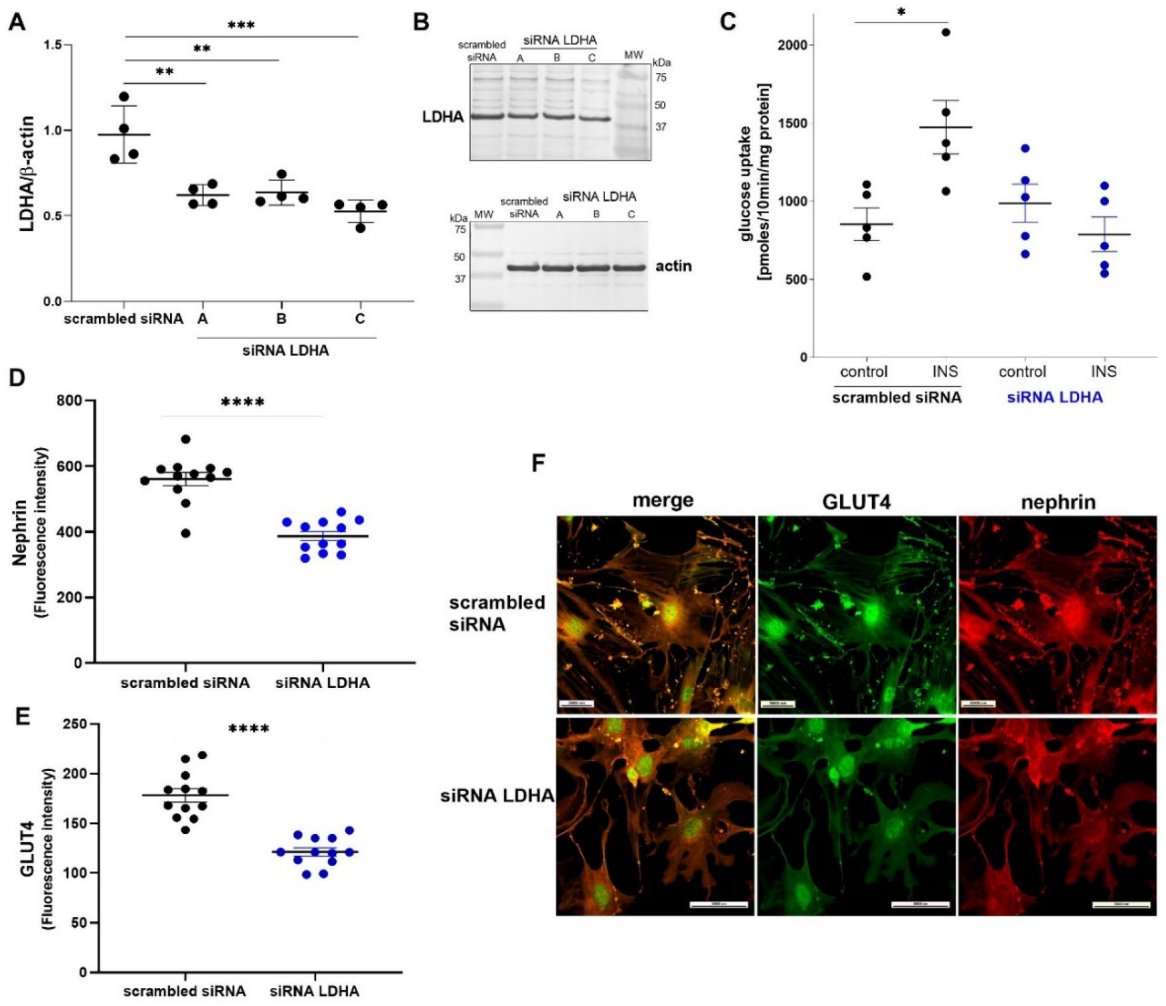
Role of LDHA downregulation in insulin-dependent glucose uptake in podocytes. (**A**) Effects of siRNA that targeted transcripts (three gene-specific siRNAs, OriGene) of LDHA in podocytes. Densitometry was performed to evaluate the expression of LDHA. Band signals were normalized to actin bands. (**B**) Representative immunoblots after the downregulation of LDHA. (**C**) Effects of LDHA downregulation on insulin-dependent increases in glucose uptake. Uptake measurements were performed after the addition of 0.5 µCi of (1,2-3* H*)-deoxy-D-glucose diluted in non-radioactive glucose to final concentrations of 50 µM and 300 nM insulin. Influence of LDHA downregulation on GLUT4 (**D**) and nephrin (F) levels and the colocalization of both proteins in primary rat podocytes (G). The data are expressed as the mean ± SEM of 4–12 independent experiments. *p < 0.05, **p < 0.01, ***p < 0.001, ****p < 0.0001.

Because HG decreased LDHA levels in podocytes, we downregulated the expression of LDHA to verify the role of this isoform in the regulation of insulin-dependent glucose uptake. Three different siRNA duplexes against LDHA were individually introduced to podocytes, and LDHA protein expression levels were assessed using Western blot. We observed a significant decrease in LDHA protein levels for all siRNA duplexes compared with cells that were transfected with scrambled siRNA (Fig. 6A). Representative immunoblots after the downregulation of LDHA we showed in Fig. 6B. We then used an siRNA duplex that induced the lowest protein expression (46% decrease for siRNA duplex C, from 0.975 ± 0.084 to 0.527 ± 0.033, *p* = 0.0001; Fig. 6A). We also observed the attenuation of stimulating effects of insulin on glucose uptake in podocytes after the downregulation of LDHA expression (Fig. 6C). Similarly, as with the inhibition of LDHA activity by oxamate, we observed a reduction of levels of GLUT4 and the key slit diaphragm protein nephrin by 32% (178.1 ± 6.8 vs. 121.4 ± 4.1, *p* < 0.00001) and 31% (560.3 ± 19.9 vs. 386.9 ± 14.4, *p* < 0.00001), respectively (Fig. 6D,E). Silencing LDHA expression also decreased the number and length of foot processes (Fig. 6F). These findings confirmed the major role of LDHA in regulating insulin-stimulated glucose uptake in podocytes.

## Discussion

**Fig. 7:**
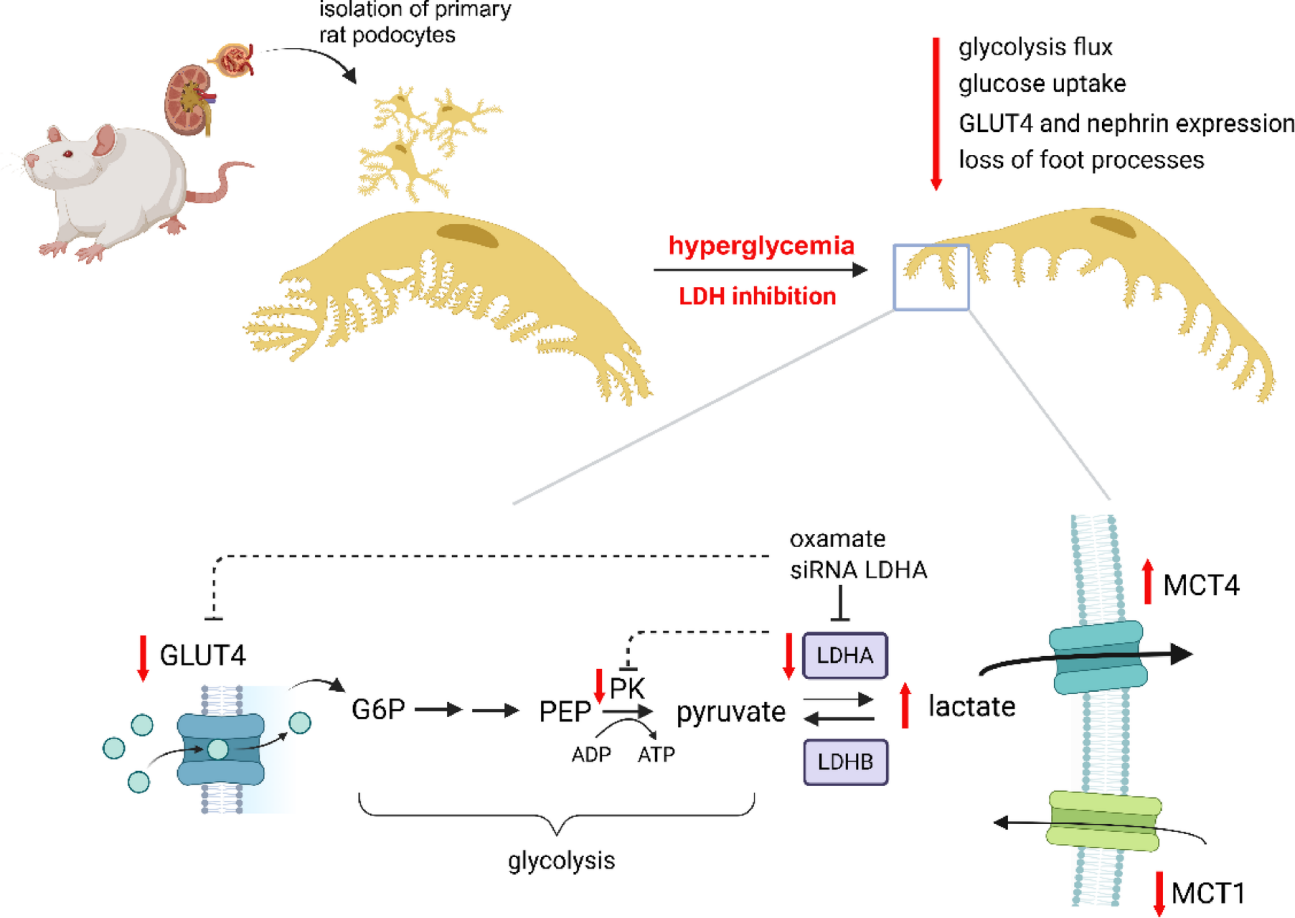
Schematic diagram of the role of LDHA in HG-induced disturbance of podocyte metabolism. Created with BioRender.com.

The present study clearly demonstrated that hyperglycemia leads to a decrease in LDH activity. We showed that low expression of the LDHA isoform is responsible for metabolic disturbances that are observed in podocytes under hyperglycemic conditions. Low LDH activity decreased glycolytic activity, affected the expression of MCTs, decreased insulin-dependent glucose uptake, and decreased the number of podocyte foot processes (Fig. 7).

Glycolysis and oxidative phosphorylation are two main cellular pathways for generating energy. Most cells can alternate between these pathways to meet varying energy demands. Research has shown that some cell types have distinctive energy metabolism systems^21^. Podocytes have been found to use both metabolic pathways to produce adenosine triphosphate^8,22^. Moreover, mitochondria were shown to be located in the cytosol around the nucleus rather than in the cortical area of podocytes^8,12^. Mitochondria were absent in foot processes of podocytes, likely because mitochondria are physically larger than foot processes. This implies that energy metabolism in foot processes relies more on glycolysis. Additionally, inhibiting glycolysis significantly decreased lamellipodia length and podocyte migratory ability^8^. Our research has shown that inhibiting LDH activity in HG or using an LDH inhibitor results in a decrease in the flow of metabolites through glycolysis, which translates to the inhibition of intracellular glucose transport and loss of podocyte foot processes. We also confirmed these relationships in cells with low expression of the LDHA isoform. These studies verify the hypothesis that energy that derives from glycolysis is essential for the proper structure of podocyte foot processes and that LDHA is an important regulator of this process.

Changes in LDH activity and the expression pattern of LDH isoforms are known to help cellular metabolism adapt to various diseases. It is commonly employed to diagnose myocardial infarction, vessel damage, tissue injury, and certain types of malignant tumors^23^. The development of diabetes was shown to be accompanied by lactic acidosis, associated with a significant increase in LDH activity in blood^24^. The authors suggested that high serum LDH levels in patients with type 2 diabetes mellitus are associated with serum levels of glycated albumin and insulin^24^. Therefore, in clinical practice, LDH can serve as an inexpensive, rapid, and easily measurable serum biomarker for assessing short-term glycemic variability.

Primary podocytes were recently shown to not depend on mitochondrial energy sources under normal, non-stressed conditions but instead convert glucose to lactate to fulfill their energy requirements^7^. This was very similar to Warburg’s hypothesis for cancer cells^25^. Moreover, HG conditions forced a human podocyte cell line to switch from oxidative phosphorylation to glycolysis, resulting in lactic acidosis^9^. Podocytes likely counteract cellular acidification, resulting from the accumulation of lactate, by its secretion into primary urine that surrounds the podocyte cell body.

In the present study, we also showed that hyperglycemic conditions and LDH inhibition increased intracellular lactate and glycogen concentrations. Lactate is one of the most common metabolites in the mammalian body. Glycolysis leads to the formation of pyruvate, part of which is converted into lactate by LDH. Lactate can also be produced from glycogen, the storage form of glucose. The plasma concentration of lactate under physiological conditions is an average of 1.5–2.5 mmol/L. Moreover, lactate has traditionally been seen as an alternative energy source to glucose. Because the lactate molecule is small, lactate is assumed to be freely filtered across the glomerular capillary. Therefore, net reabsorption of this metabolite must occur along the nephron. The kidney is active in the uptake of monocarboxylates from the glomerular filtrate against energy loss and for gluconeogenesis; thus, the reabsorption rate of lactate in the mammalian kidney is very high (> 95%)^26,27^.

Lactate is transported via two types of transporters that belong to the solute carrier family (SLC) and are selective for monocarboxylates: proton-coupled MCTs and sodium-coupled monocarboxylate transporters (SMCTs). SMCTs comprise SMCT1 (SLC5A8) and SMCT2 (SLC5A12). In the kidney in mammals, the low-affinity Na^+^-lactate cotransporter SMCT2 mediates the bulk of lactate reabsorption in the early part of proximal convoluted tubules (S1), whereas the high-affinity SMCT1 in the distal part of proximal tubules (S2-S3) reduces lactate in urine to very low levels^27,28^.

These transporters play an important role in pH regulation. Moreover, kinetic activities of MCT1 (K_m_ = 3.5–10 mM) and MCT4 (K_m_ = 22 mM) are quite different. MCT1 expression was shown to be highly correlated with oxidative fiber composition of the muscle and other indices of oxidative metabolism. Lactate uptake from the circulation is also highly correlated with MCT1 content in muscles. MCT4 is confined to fast-twitch (fast glycolytic and fast oxidative glycolytic) muscle fibers, in which MCT4 content is correlated with indices of anaerobic metabolism. Collectively, these data suggest that MCT1 and MCT4 are responsible for lactate uptake from the circulation and lactate extrusion out of muscle, respectively^16^.

We recently characterized the lactate transport system in podocytes. We demonstrated that lactate regulates expression patterns of these transporters in podocytes that are cultured in the presence of a SG concentration or limited glucose availability^11^. The present study demonstrated that HG increases MCT1 and MCT4 levels in the cell membrane. Additionally, we showed that HG decreased MCT1 expression and increased MCT4 expression in podocytes. We observed the same effect after the inhibition of LDH activity by oxamate. This indicates an increase in expression of the transporter that is responsible for lactate efflux from the cell, which is observed in cells with enhanced glucose metabolism.

A previous study demonstrated that Zucker rats (i.e., a model of type 2 diabetes) with high plasma insulin levels had high plasma lactate concentrations and low muscle MCT4 and MCT1 content compared with control rats^29^. In streptozotocin-induced diabetes (i.e., a model of type 1 diabetes), resting blood lactate was reported to be elevated^30^, and streptozotocin‐induced diabetes in rats has also been found to decrease skeletal muscle lactate transport^30,31^. Moreover, a study in streptozotocin‐induced diabetic rats revealed a selective reduction of MCT1 and MCT4 density in some skeletal muscles and in the heart^31^. All these studies indicate that hyperglycemia affects the distribution of these transporters. Our new and significant finding is that low LDH activity is responsible for changes in the amount of MCTs in the presence of HG concentrations.

Lysine lactylation is a recently discovered post-translational modification that depends on lactate. It was first identified in histones by Zhang et al. in 2019 and has since been implicated in various pathological processes, including cancer, metabolism, and inflammatory diseases^32^. Moreover, it has been shown that treatment of cells with glycolytic inhibitors, including 2-deoxy-D-glucose and oxamate, which reduced lactate accumulation, leads to a decrease in histone lactylation^32^. Resent study identified a significant link between lysine lactylation and diabetic nephropathy^33^. Biopsy samples from patients with diabetic nephropathy and db/db mice (a model of type 2 diabetes) showed elevated lysine lactylation levels compared to controls. Notably, lactylation at K182 of ACSF2 (Acyl-CoA Synthetase Family Member 2) led to mitochondrial dysfunction^33^. Another study demonstrated that treating primary human skeletal muscle cells with lactate resulted in a dose-dependent increase in protein lactylation and IRS-1 serine 636 phosphorylation, a modification previously linked to insulin resistance^34^. Therefore, increased lactate accumulation in diabetes may further negatively affect kidney function by enhancing protein lactylation. Targeting this process could offer a potential therapeutic approach for diabetic complications.

In summary, anaerobic glycolysis and the fermentation of glucose to lactate are part of one of the major metabolic pathways in podocytes. We postulate that the decrease in LDH activity is responsible for the inhibition of glucose uptake and glycolysis. Furthermore, our findings demonstrate that the LDHA isoform is responsible for low LDH activity in hyperglycemia. This highlights the important role of this LDH isoform in the adaptive response of cellular metabolism to elevated glucose levels that are associated with diabetes.

## Materials and methods

### Ethics statement and glomeruli isolation

Experiments were performed with Wistar male rats that were obtained from the Mossakowski Medical Research Institute, Polish Academy of Sciences, Warsaw, Poland. The rats were maintained on a 12 h light/12 h light/dark cycle with free access to a standard pellet diet and tap water. All experimental procedures involving animals were reviewed and approved in compliance with Directive 2010/63/EU of the European Parliament and of the Council on the protection of animals used for scientific purposes. The ARRIVE guidelines were employed for reporting experiments involving live animals, promoting ethical research practices. All experiments were conducted in compliance with the guidelines of University of Gdańsk (4/D000/2024).

We used female Wistar rats, weighing 100–120 g. After immobilizing the animal by dorsal grip, they were administered with a mix of ketamine (65 mg/kg body weight) and xylazine (5 /mg/kg body weight) intraperitoneally. The kidneys were excised and minced with a scalpel and then pressed through a system of sieves with a decreasing pore diameter (160, 106, and 53 μm) to obtain a suspension of glomeruli in RPMI 1640 supplemented with 10% FBS and 100 U ml^− 1^ of penicillin with 100 µg ml^− 1^ streptomycin. The final suspension of glomeruli was plated to 75 cm^2^ type I collagen-coated culture flasks (Becton Dickinson Labware, Beckton, UK) and maintained at 37 °C in an atmosphere of 95% air and 5% of CO_2_ for 5–7 days.

### Preparation and culture of primary rat podocytes

Podocytes were isolated as described previously^35^. Experiments were conducted using podocytes that were cultivated for 12–20 days. Cell phenotypes were established using podocyte-specific antibodies against Wilms tumor-1 protein (Biotrend, Koeln, Germany) and synaptopodin (Progen, Heidelberg, Germany).

Podocytes were cultured in standard medium (RPMI 1640, Sartorius, catalog number 01-100-1 A) that contained a standard glucose (SG) concentration (11.1 mM, used as the control condition) and a high glucose (HG) concentration (30 mM) for 5 days when we observed the induction of insulin resistance^36^. Both control cells and cells with HG were supplemented with the LDH inhibitor oxamate (45 mM, Sigma-Aldrich) for 24 h.

### Real-time polymerase chain reaction

Total RNA from cultured primary rat podocytes was isolated using the RNeasy Mini Kit (Qiagen, Hilden, Germany), along with the RNase-Free DNase Set (Qiagen, Hilden, Germany) to remove genomic DNA. The quantity and purity of isolated RNA were assessed by spectrophotometric measurements (NanoDrop 2000; Thermo Fisher Scientific, Waltham, MA, USA). A reverse transcription reaction and real-time polymerase chain reaction (PCR) were performed as described previously^37^. Nucleotide sequences of gene-specific primers and Taqman probes are listed in Table 1. mRNA expression levels of the *β-actin* gene served as a control for cDNA load. To verify the proper molecular weight of PCR end-products, each one was separated in 2.5% agarose gel and imaged in a Gel Doc XR + System (Bio-Rad, Hercules, CA, USA).

**Table 1:** Primers and probes used in the real-time PCR experiments.

Gene	Accession no.	Primer sequence (5’-3’)	Probe (5’-3’)	Product
*LDHA*	NM_017025.2	Forward: ACCACGCACTTCTCATCTGAGC Reverse: GTGAGGGTGCGTAGCACAGC	FAM-CTGTCTCC-BHQ1	215 bp
*LDHB*	NM_001316333.1	Forward: TTGTCTGGACAAGATGGCAAC Reverse: TCAGCCACGATTTTCGGAGT	FAM-TGCCCTGG-BHQ1	255 bp
*β*-*Actin*	NM_031144.3	Forward: CATCCTGACCCTGAAGTA Reverse: TGCCAAATCTTCTCCATATC	HEX-CACGGCATTGTCACCAAC-BHQ1	72 bp

### Western blot

The protocols for cell lysate preparations and Western blot analyses were described previously^11^. Primary antibodies are listed in Table 2. The densitometric analyses were performed using Quantity One software (Bio-Rad, Hercules, CA, USA).

**Table 2:** Primary antibodies used in the experiments.

Antigen	Clonality	Dilution	Source	Catalog no.
LDHA	Polyclonal	1:2000	ABclonal	A1146
LDHB	Monoclonal	1:500	Santa Cruz Biotechnology	sc-133,123
MCT1	Polyclonal	1:400	Merck Millipore	AB1286-I
MCT4	Polyclonal	1:1250	Biorbyt	orb524984
actin	Monoclonal	1:10000	Sigma-Aldrich	A5441

### Cell surface biotinylation

The biotinylation of cell membrane proteins was performed as previously described^38,39^. Briefly, podocytes were incubated with 1 mg/ml biotin solution (catalog no. 21338, Thermo Fisher Scientific Inc., Waltham, MA, USA) for 30 min at 4 °C. Next, cell lysates were generated and then incubated with Neutr/Avidin resin (catalog no. 53150, Thermo Fisher Scientific Inc., Waltham, MA, USA) on a rotor at 4 °C overnight. The next day, membrane samples (which bound to Avidin) and total samples were subjected to Western blot analysis.

### Fluorescent staining and bioimaging analysis

Podocytes were cultured on microscopy cell culture Petri dishes (Ibidi, Planegg/Martinsried, Germany). Cells were fixed in PBS containing 4% formaldehyde for 15 min at room temperature. Fixed podocytes were permeabilized with 0.1% Triton-X for 2–3 min and then blocked with PBSB solution (phosphate-buffered saline [PBS] plus 2% fetal bovine serum [FBS], 2% bovine serum albumin, and 0.2% fish gelatin) for 1 h. After blocking, the cells were incubated with anti-LDHA (1:200, ABclonal, catalog no. A1146), anti-LDHB (1:100, Santa Cruz Biotechnology, catalog no. sc-133123), anti-monocarboxylate transporter 1 (MCT1; 1:50, Merck Millipore, AB1286-I), anti-MCT4 (1:50, Biorbyt, orb524984), anti-glucose transporter type 4 (GLUT4; 1:100, Santa Cruz Biotechnology, catalog no. sc-53566), and anti-nephrin (1:200, Sigma-Aldrich, catalog no. PRS2265) antibodies in PBSB at 4 °C for 2 h. Next, the cells were washed three times with cold PBS and incubated with appropriate secondary antibodies that were conjugated to Alexa Fluor 488 (1:200) or Alexa Fluor 546 (1:200). Fluorescence imaging was performed using a confocal microscope (Eclipse Ti, Nikon Instruments, Melville, NY, USA; RCM device, Confocal.nl, Amsterdam, Netherlands). The mean fluorescence intensity of the outlined regions of interest was measured using Nikon NIS-Elements software with the Automated Measurement Results option.

### Measurement of extracellular acidification rate

Podocytes were seeded on eight-well culture microplates (Agilent, Santa Clara, CA, USA), coated with Rat Tail Collagen-I Solution (Sigma Aldrich, St. Louis, MO, USA), and were cultured for 12–14 days. On the day of an experiment, growth medium was replaced with assay medium (minimal Dulbecco’s Modified Eagle Medium supplemented with 2 mM L-glutamine), and the plates were kept at 37℃ without CO_2_ for 45 min. The plates were then transferred to a Seahorse XFp analyzer (Agilent, Santa Clara, CA, USA), and extracellular acidification rate (ECAR) values were determined before and after the injection of 10 mM D-glucose, 1 µM oligomycin, and 50 mM 2-deoxy-D-glucose. Baseline and post-exposure rates were measured three times every 3 min (total analysis time: 70 min). The glycolytic flux parameters were determined from slopes of ECARs in real-time analyses using Wave 2.6.3.5 software (Agilent, Santa Clara, CA, USA). The ECARs were normalized to protein concentrations that were determined for each culture-plate well using the Bradford method.

### Glucose uptake assay

Glucose uptake was measured as described previously with minor modifications^40^. Cells were washed three times with warmed Ca^2+^- and Mg^2+^-free PBS and incubated for 2 h with serum- and glucose-free RPMI 1640 medium. Glucose uptake was measured using 0.5 µCi/well of (1,2-3* H*)-deoxy-D-glucose diluted in non-radioactive glucose at a final concentration of 50 µM with or without 300 nM insulin for 10 min. Radioactivity was measured by liquid scintillation counting using a MicroBeta2 Microplate Counter (Perkin Elmer, Waltham, MA, USA).

### siRNA interference and cell transfection

Podocytes were transfected with small-interfering RNAs (siRNAs) that targeted LDHA (three unique 27mer siRNA duplexes, catalog no. SR507071, OriGene Technologies, Rockville, MD, USA) or with control, non-silencing siRNA (negative control; OriGene Technologies, Rockville, MD, USA). One day before the experiment, the culture medium was replaced with antibiotic-free RPMI‐1640 that was supplemented with 10% FBS. For podocyte transfection, Lipofectamine 3000 was used in combination with Opti‐MEM (Gibco, Thermo Fisher Scientific Inc., Waltham, MA, USA), and siRNA was also dissolved in Opti-MEM. Transfection was performed in accordance with the manufacturer’s instructions. Transfection reagent and siRNA were incubated with Opti-MEM for 5 min and then combined together for 15 min of incubation. Complexes of siRNA and transfection reagent were gently added to cell cultures and incubated at 37 °C. After 7 h, a medium with a 2× higher concentration of FBS and antibiotics was added to the podocytes and incubated for the next 24 h at 37 °C, and then the culture medium was replaced with standard RPMI‐1640. Gene silencing was examined by Western Blot.

### Determination of lactate dehydrogenase and pyruvate kinase activities

All enzyme activities were determined according to Bergmeyer^41^ with minor modifications^11^. All activities were measured in 50 mM Tris/HCl (pH 8.0) that contained 50 mM KCl, 1 mM MgCl_2_, and 0.2 mM NADH or 0.4 mM NADP, respectively. Lactate dehydrogenase activity was determined with 2.5 mM pyruvate. Pyruvate kinase activity was determined with 5 mM phosphoenolpyruvate, 2 mM adenosine diphosphate, and 5 U/ml LDH (Sigma-Aldrich). Cells were lysed in buffer that was supplemented with a proteinase inhibitor cocktail (Sigma Aldrich) and 0.05% Triton X-100. The reaction was started by the addition of cell lysate (10 µg protein) to the assay buffer. Measurements were performed using a Lambda 265 UV/VIS spectrophotometer (Perkin Elmer, Waltham, MA, USA) at an absorbance of λ = 360 nm.

### Lactate, pyruvate, and glycogen concentrations

Lactate, pyruvate, and glycogen concentrations were measured in lysed podocytes using the Lactate Assay Kit, Pyruvate Assay Kit, and Glycogen Assay Kit (Sigma-Aldrich, Merck KGaA, St. Louis, MO, USA).

### Statistical analysis

All statistical analyses were performed using GraphPad Prism 8 software. The Shapiro-Wilk test was used to determine whether parametric or nonparametric tests should be conducted. The statistical analyses were performed using one-way analysis of variance. The data are expressed as the mean ± SEM. Values of *p* < 0.05 were considered statistically significant.

## Electronic supplementary material

Below is the link to the electronic supplementary material.

Supplementary Material 1

## Data Availability

All datasets generated and/or analysed during the current study available from the corresponding author on reasonable request.
