# Supplementary material for: Role of lactate dehydrogenase A in the regulation of podocyte metabolism and glucose uptake under hyperglycemic conditions
Source: Sci Rep. 2025 Apr 23;15:14162. doi: 10.1038/s41598-025-98797-0 (PMC12019540; doi:10.1038/s41598-025-98797-0)

Figure 1:

LDHA

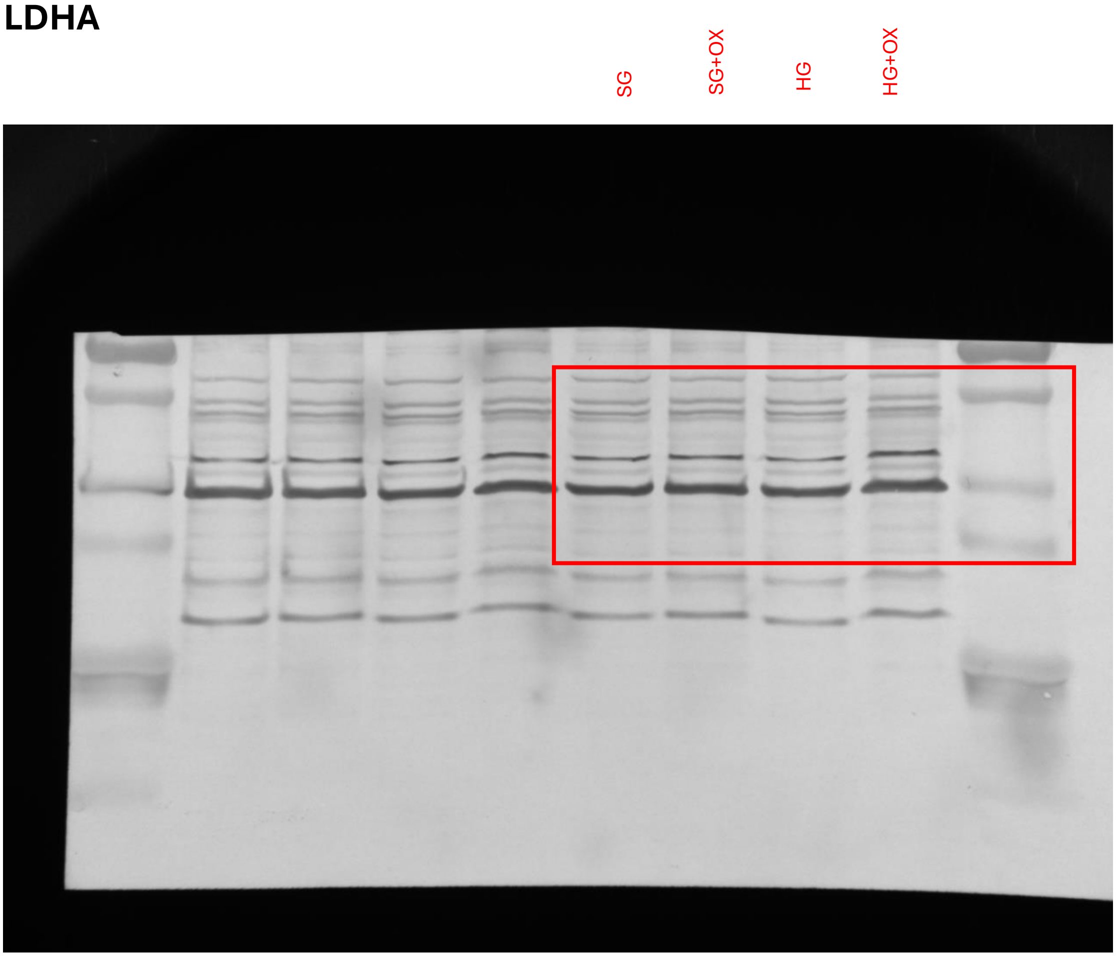

LDHB

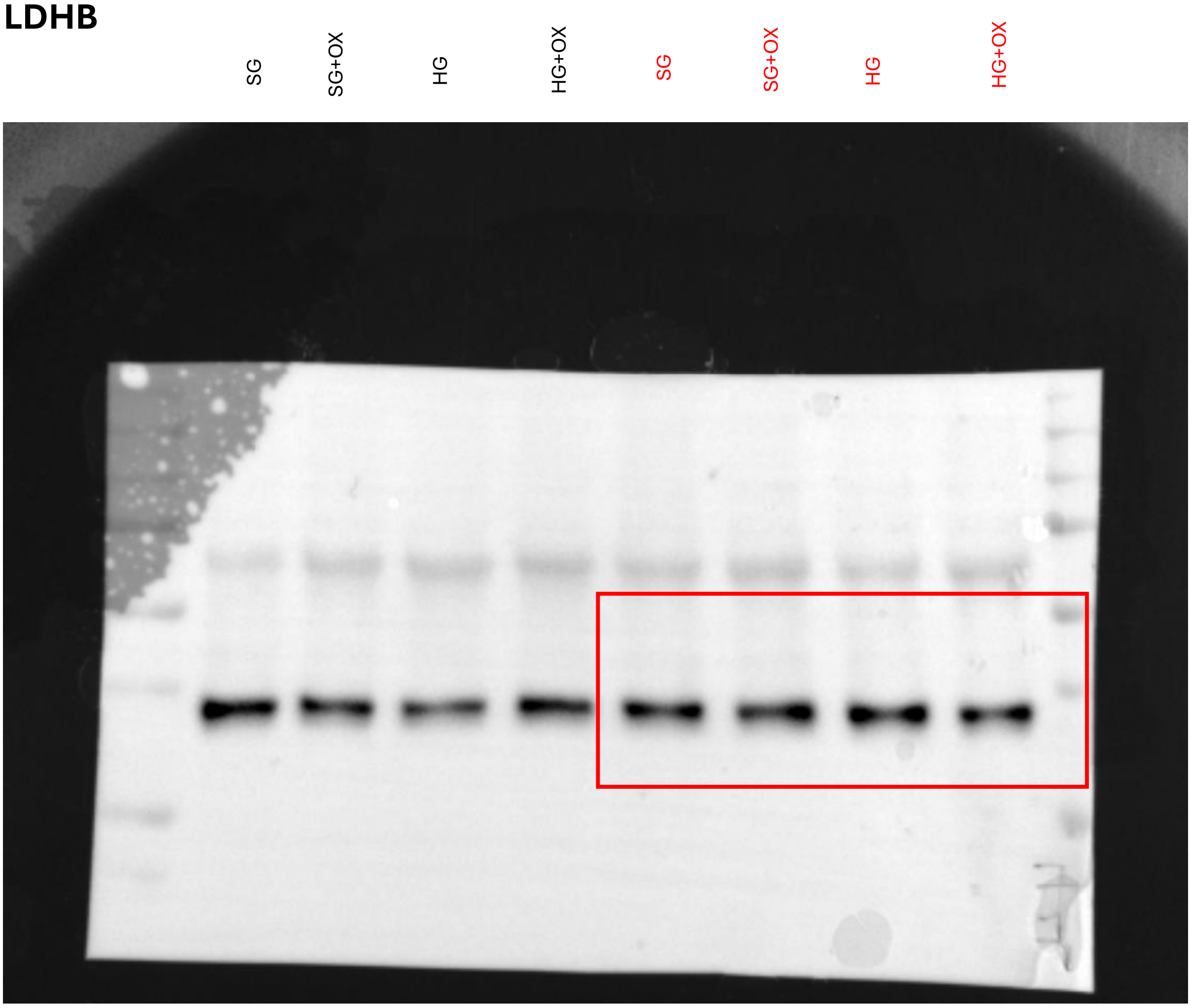

Actin

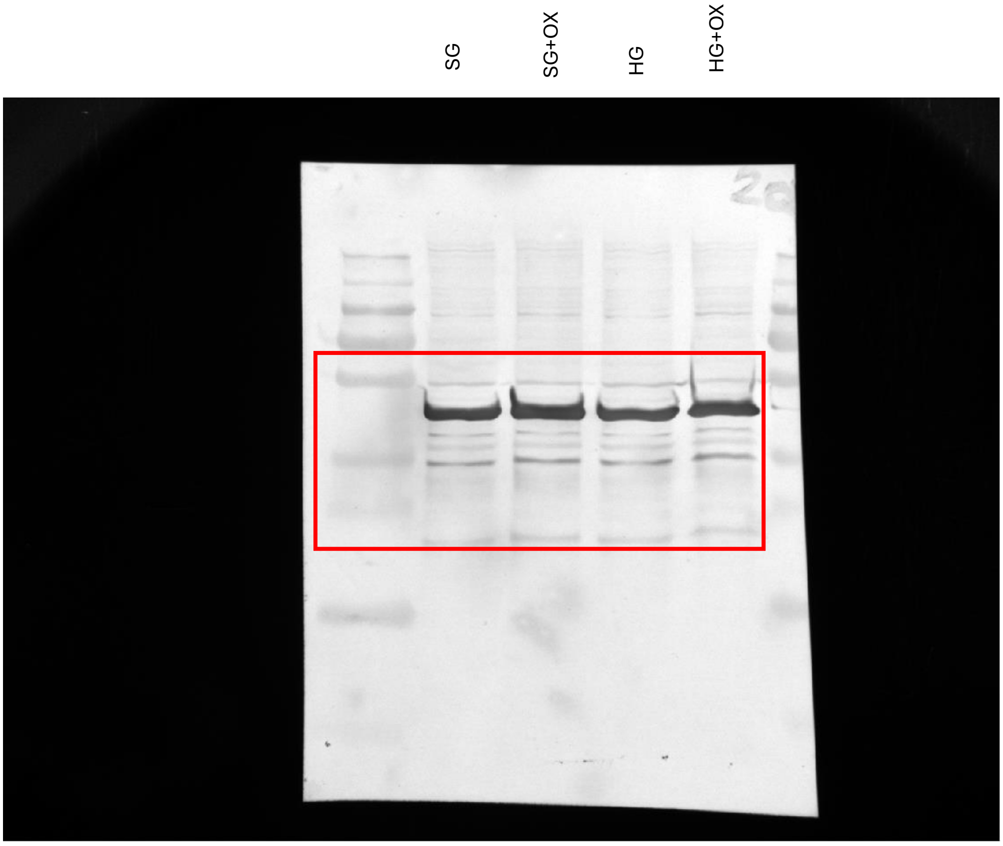

Figure 2:

actin

SG SG+OX HG HG+OX

37 kDa

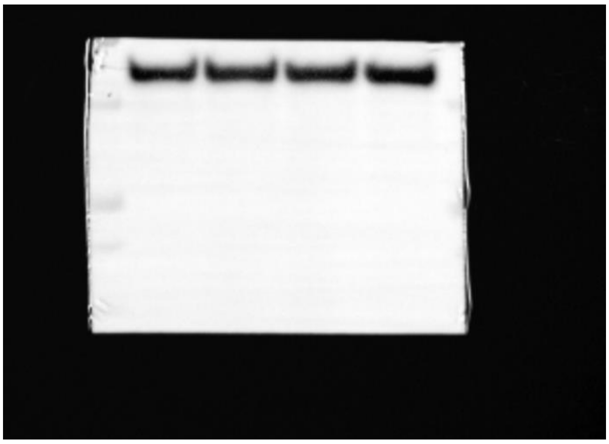

MCT4

SG SG+OX HG HG+OX SG SG+OX HG HG+OX

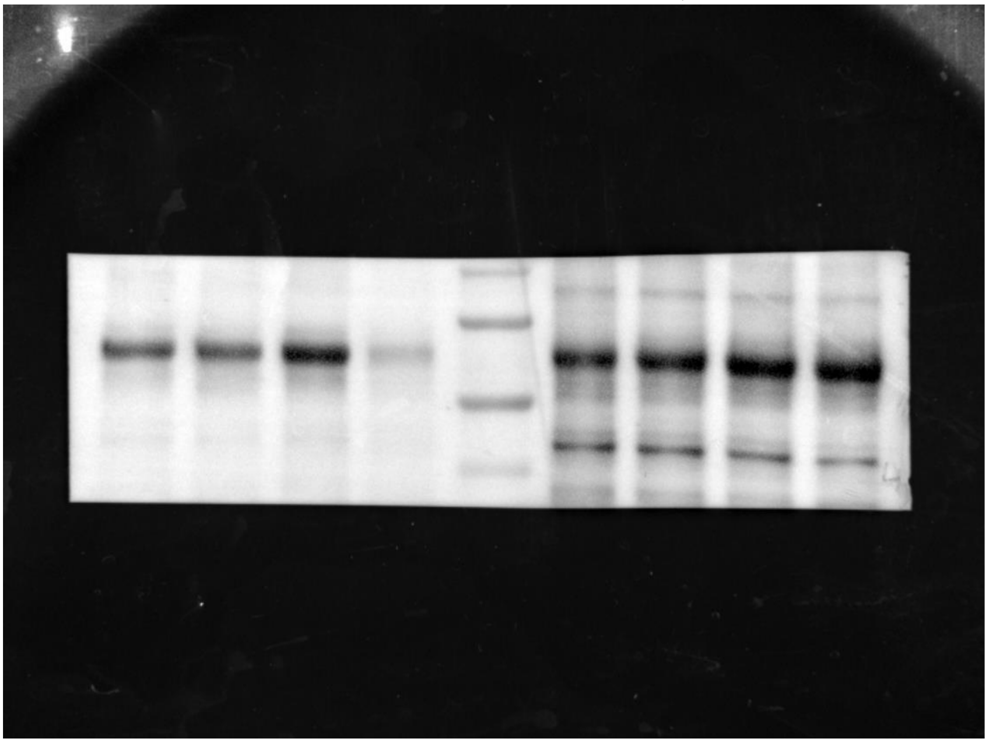

MCT1 membrane

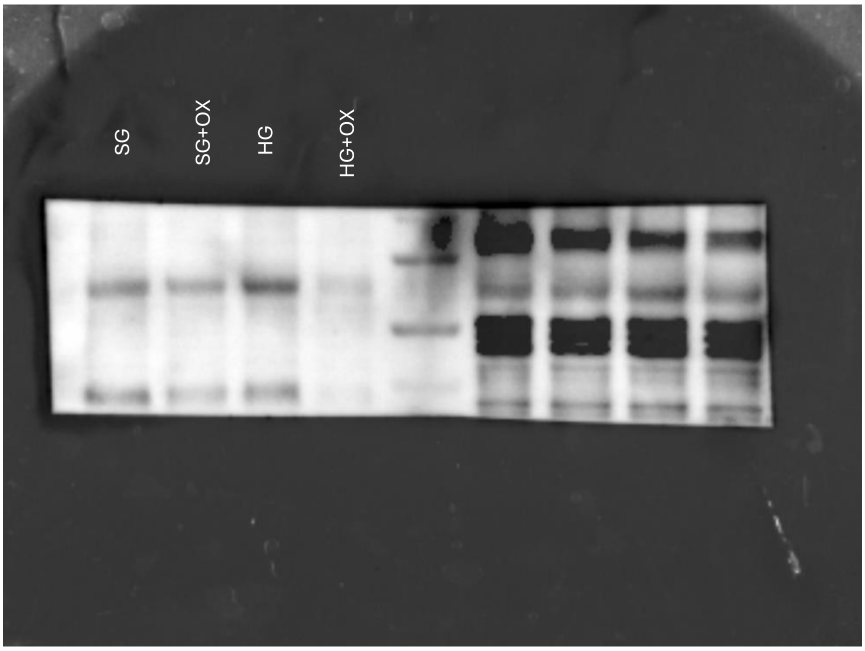

MCT1 total

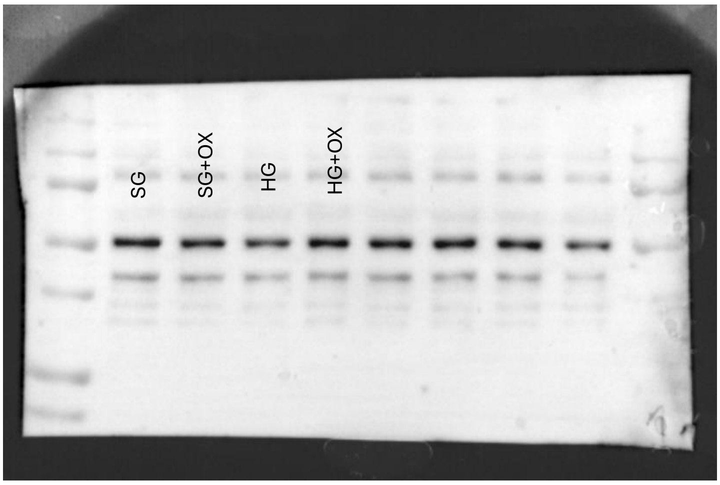

Figure 6:

LDHA

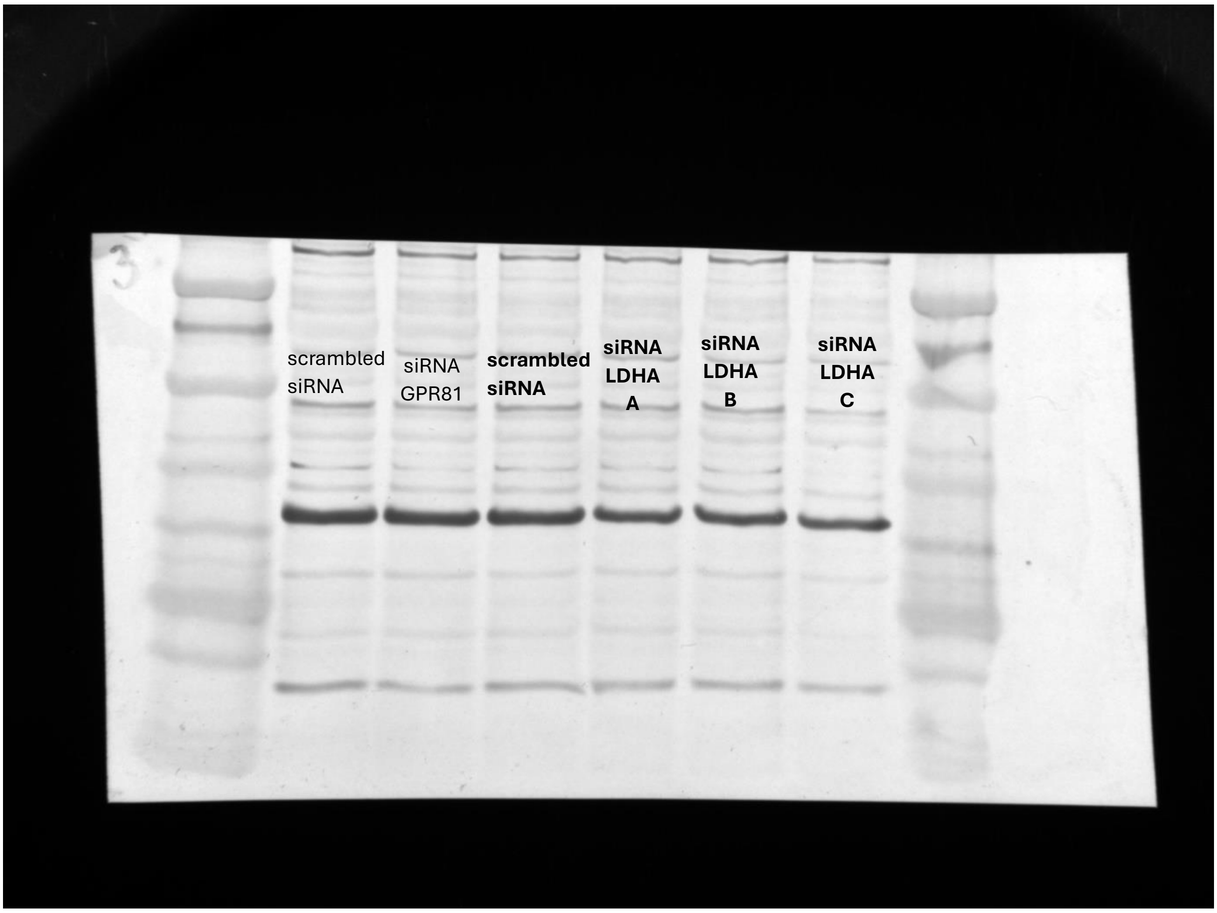

actin

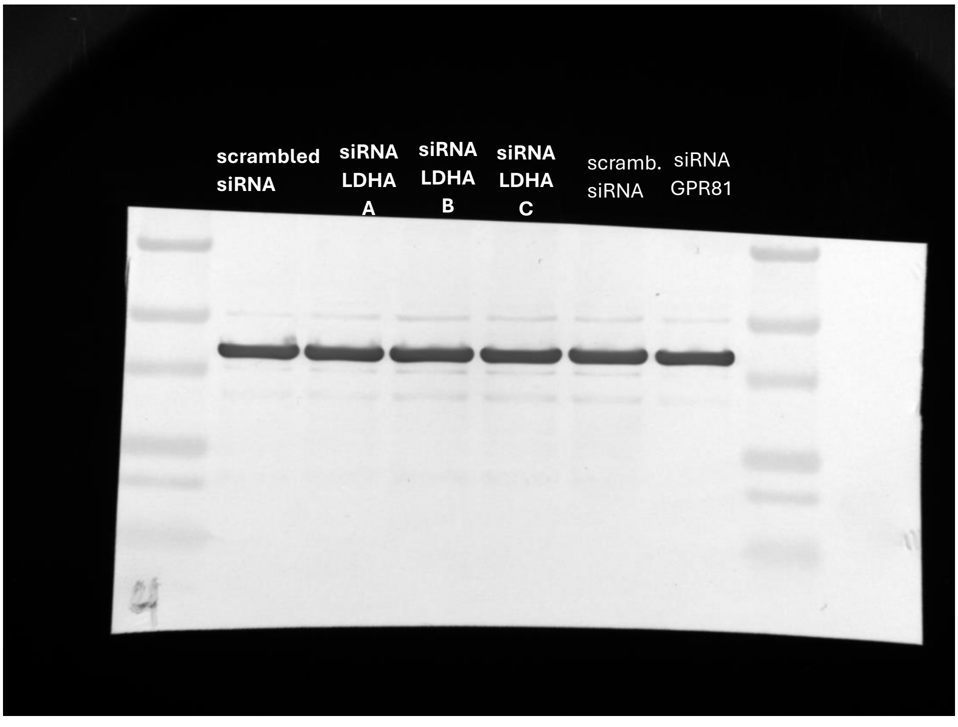

Supplement: Supplementary file 1 — Supplementary Material 1 [file 41598_2025_98797_MOESM1_ESM.pdf]
